# Toxicity and synergistic activity of compounds from essential oils and their effect on detoxification enzymes against *Planococcus lilacinus*

**DOI:** 10.3389/fpls.2022.1016737

**Published:** 2022-10-24

**Authors:** Charles Arokiyaraj, Kangkanjyoti Bhattacharyya, Sajjalavarahalli G. Eswara Reddy

**Affiliations:** ^1^ Entomology Laboratory, Agro-Technology Division, CSIR-Institute of Himalayan Bioresource Technology, Palampur, India; ^2^ Academy of Scientific and Innovative Research (AcSIR), Ghaziabad, India

**Keywords:** mealybug, essential oil compounds, contact toxicity, synergistic, AChE, GST, scanning electron microscopy

## Abstract

Mealybug, *Planococcus lilacinus* Cockerell, is a primary surface-feeding insect pest of fruit and flowering plants and also transmits plant viruses, resulting in economic crop loss. Inappropriate and recurrent use of pesticides for mealybug control results in resistance building and deleterious effects on humans and the environment. Essential oils are the most excellent choice for insecticides. Insecticidal activities of pure compounds of essential oils against *P. lilacinus* are not reported. The present study aims to study the insecticidal activities of some pure active compounds and their binary mixture’s action by topical application against *P. lilacinus*. Results showed that the pure compounds of L-limonene, β-myrcene, and ocimene revealed toxicity (each at LD_50_ = 0.37 µg/insect) after 96 h. The binary mixtures of geraniol + L-menthol and L-limonene + geraniol exhibited synergistic effects (each at LD_50_ = 0.03 µg/insect) after 96 h. The monoterpenes of ocimene and β-myrcene at the higher concentration of 5,000 ppm substantially inhibited the detoxification enzyme activities of AChE (0.93 and 0.78 mU/mg, respectively) and GST (2.19 and 7.29 nmol/min/ml, respectively) in *P. lilacinus* after 48 h. SEM analysis reported the significant anomalies on the morphology of abdominal cuticle, setae, and thoracic leg after 96-h treatment with ocimene at 1,250 ppm against *P. lilacinus*. Based on the results, the tested pure compounds and their combinations can be suggested for the control of mealybugs.

## Introduction

Mealybug, *Planococcus lilacinus* Cockerell (Hemiptera: Pseudococcidae), is a major surface-feeding pest of many fruits and flowering plants (Huang et al., 2021; Ren et al., 2022). It is also regarded as a major pest that causes severe damage to various commercially significant crops, including citrus, coconut, coffee, custard apple, dragon fruit, guava, grape, mango, and tamarind, leading to significant economic yield loss (Nguyen et al., 2016). Adults and crawlers can be found in the leaf nodes, flower sepals, and tree trunk crevices, easily carried through global trade. *P. lilacinus* infestations are indicated by stunting, yellowing, distortion of leaves, defoliation, clumping of shoots, thickening of stems, and transmission of plant viruses. Moreover, the honeydew of *P. lilacinus* released on the leaves promotes the development of black sooty molds, obstructing photosynthesis and lowering the crop yield of sugar apples (Jahn, 1993). The outer layer of *P. lilacinus* is extensively coated with wax constituted by the lipids that make them difficult to control.

The current management of mealybug control is being carried out using a broad spectrum of synthetic pesticides of various types. However, the indiscriminate and recurrent use of pesticides against the mealybugs is unsustainable due to environmental degradation, biodiversity loss, and resistance development (Fouad et al., 2016). However, studies have reported mealybugs with the resistance development to many well-known classes of pesticides, including avermectins, carbohydrazide, cyclodiene, IGRs, neonicotinoids, organophosphates, oxadiazine, and pyrethroids in recent times (Saddiq et al., 2014; Nagrare et al., 2020). Owing to the adverse impact of the chemical pesticides on the public and the environment, an immediate replacement is required to successfully control the mealybugs. Plant-derived botanical agents such as extracts and essential oils from non-host plants with repellent, antifeedant, and toxicant properties may be considered as a better alternative for chemical pesticides for the control of pests (Regnault-Roger et al., 2012).

Several plant-derived essential oils of *Acorus calamus*, *Aegle marmelos*, *Cedrus deodara*, *Mentha piperita*, *Mentha spicata*, *Murraya koenigii*, and *Tagetus minuta* were reported to have insecticidal activities against various insect pests in previous studies (Reddy et al., 2016; Jayaram et al., 2022a). Additionally, the homogeneous or heterogeneous blends of essential oils also revealed that the insecticidal activities against various species of mealybugs, including *Pseudococcus calceolariae* (Tacoli et al., 2018), *Planococcus citri* (Cloyd et al., 2009; Erdemir and Erler, 2017), *Planococcus ficus* (Karamaouna et al., 2013; Peschiutta et al., 2017; Tacoli et al., 2018; Brahmi et al., 2022), *Pseudococcus longispinus* (Hollingsworth and Hamnett, 2009; Tacoli et al., 2018), *Paracoccus marginatus* (Mwanauta et al., 2021), *Planococcus minor* (Prabowo and Damaiyani, 2019), *Phenacoccus solenopsis* (Mostafa et al., 2018; Saad et al., 2021), and *Pseudococcus viburni* (Ramzi et al., 2017) have been reported earlier. Essential oils often consist of a mixture of different biological compounds or phytochemicals with varied polarity, and separating them is still a significant challenge (Sasidharan et al., 2011). Henceforth, pure compounds commercially available have been widely used to study the insecticidal activities. To our knowledge, the toxicity of pure compounds from the plant essential oils against *P. lilacinus* is yet to be studied. Hence, exploring some plant-derived pure compounds from the various essential oils is possible in the present investigation to minimize the reliance on chemical pesticides and mode of action to control mealybugs. Based on this context, the primary objectives of this study are to investigate the selected pure compounds and their combinations for their toxicity, synergistic and detoxification enzyme inhibition activities, and morphological changes against the crawlers of *P. lilacinus* to find the inevitable lead(s) for botanicals production.

## Materials and methods

### Chemicals

**Figure 1:**
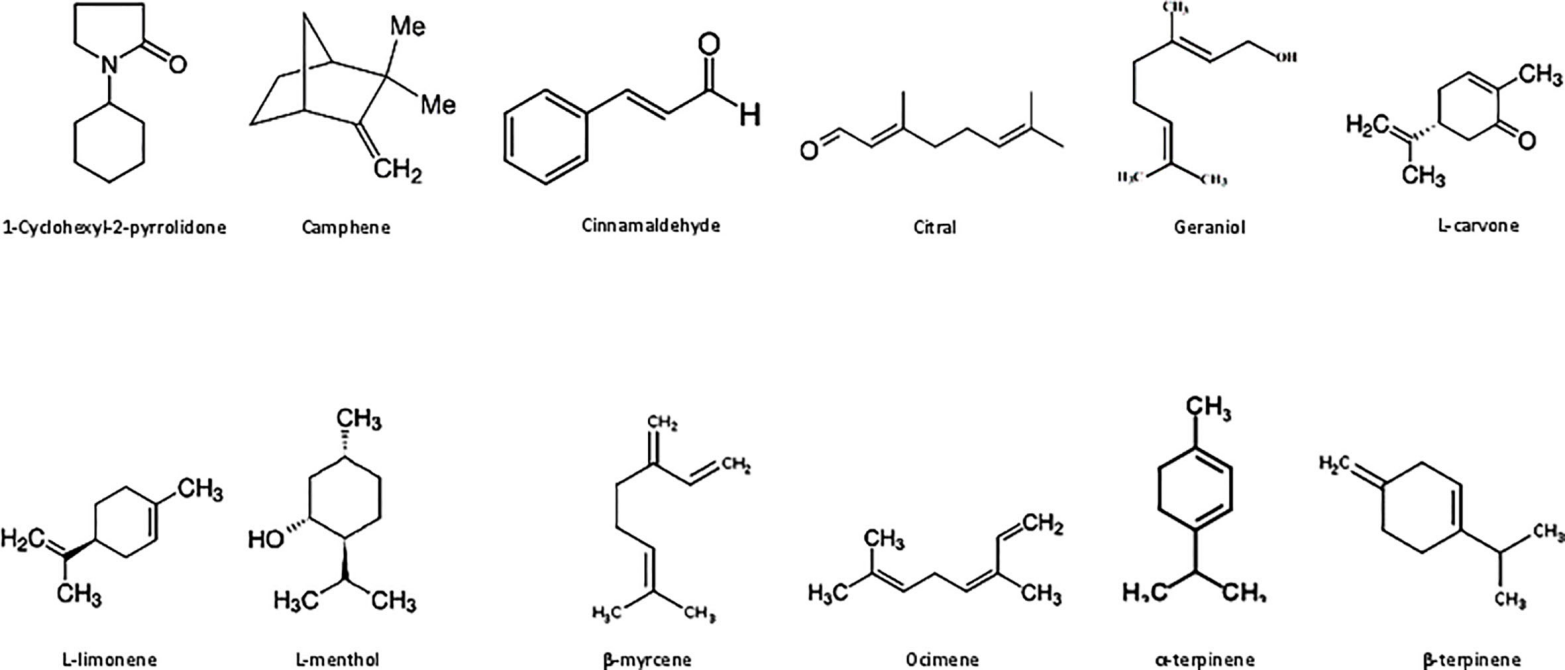
Some active pure compounds from essential oils.

Phytochemicals such as 1-cyclohexyl-2-pyrrolidone, camphene, cinnamaldehyde, citral, geraniol, L-carvone, L-limonene, L-menthol, β-myrcene, ocimene, α-terpinene, and β-terpinene (**Figure 1**) and other essential chemicals were purchased from Sigma-Aldrich Chemicals Pvt. Ltd., India.

### Experimental insect maintenance

*P. lilacinus* were collected from the outdoor area and consistently reared on live tobacco plant, *Nicotiana tabacum* L., in the insectary unit of Agrotechnology Division, CSIR-IHBT, Himachal Pradesh, under regulated temperature (25 ± 2°C), relative humidity (60 ± 5%), and a 16:8 light–dark environment for several generations. The fresh second instar crawlers were used for toxicity bioassays.

### Comparative toxicity of pure compounds

The toxicity of pure compounds such as 1-cyclohexyl-2-pyrrolidone, camphene, cinnamaldehyde, citral, geraniol, L-carvone, L-limonene, L-menthol, β-myrcene, ocimene, α-terpinene, and β-terpinene was initially evaluated against the second instar crawlers of *P. lilacinus* as per the standard topical application method (Machial et al., 2010) for LD_50_ determination. The commercially available botanical formulation (Neem Baan contains azadirachtin 1,500 ppm) for the control of sucking pests was used as a positive control for comparison. Briefly, five different concentrations (5,000, 2,500, 1,250, 625, and 312.5 ppm) of pure compounds and Neem Baan were prepared by blending each compound in 0.05% Tween-80. One microliter of sample was administered using a Hamilton micro-syringe with a repeating dispenser dorsally to each of 10 second instar crawlers of *P. lilacinus.* Treated crawlers were then transferred to tea leaf discs (3 cm^2^) in each Petri plate, pressed on top of the water-agar medium to preserve the greenness of leaf discs. Five replications were maintained for each treatment and kept under controlled conditions for observations. The mortality of the crawlers was documented after 24, 48, 72, and 96 h of treatment.

### Toxicity of binary mixtures of pure compounds

Based on the toxicity study, the blends/mixtures of pure compounds such as ocimene + β-myrcene, ocimene + L-limonene, ocimene + geraniol, ocimene + L-menthol, β-myrcene + L-limonene, β-myrcene + geraniol, β-myrcene + L-menthol, L-limonene + geraniol, and geraniol + L-menthol were prepared in five concentrations and proportions, as a 1:1 ratio of their individual LD_50_ values, for the bioassay and synergistic activity against *P. lilacinus* in the lab environment. Samples were prepared and administered against the second instar crawlers of *P. lilacinus* as mentioned above. Each treatment was performed using five replications, and the mortality of the crawlers was documented at 24, 48, 72, and 96 h after treatment for the combined compounds to calculate the LD_50_ and cotoxicity coefficient (CTC) values (Sun and Johnson, 1960). The CTC was determined by the following equation: CTC = [LD_50_ of compound/LD_50_ of the compound in a combination] * 100. If the combination provides a CTC greater than 100, a synergistic action is exhibited; CTC less than 100, individualistic action; and CTC equal to 100, similar action.

### Detoxification enzyme inhibition of pure compounds against *P. lilacinus*

#### Sample preparation

Inhibition activities of detoxification enzymes [acetylcholinesterase (AChE) and glutathione-S-transferase (GST) were carried out using standard procedures (Chauhan et al., 2022). Based on the toxicity assay report, four different concentrations of ocimene and β-myrcene (5,000, 2,500, 1,250, and 625 ppm) were prepared for detoxification enzyme inhibition activity. After 24 and 48 h of treatment, 20 mg of survived crawlers was taken for each concentration and was subjected to detoxification enzyme inhibition analysis. The crawlers for each concentration were collected in the centrifugation tube and subjected to homogenization using phosphate buffer (0.1 M; pH 7.4) at a 1:9 ratio in the micropestle (Tarsons). The homogenate was then promptly shifted to fresh tubes under ice bath conditions and centrifuged at 12,000 rpm for 30 min at 4°C. The clear supernatant was taken for storage at −20°C for subsequent enzyme studies.

#### Protein estimation

xProtein estimation was analyzed following the standard protocol (Bradford, 1976) before proceeding with the detoxification enzyme inhibition assay. Briefly, adding 5 µl of treated and untreated mealybug homogenates (obtained from each concentration) with 35 µl of MilliQ water in 160 µl of Bradford reagent in triplicate followed by the average values was carried out for protein estimation. The blend was incubated at room temperature (RT) for 15 min. After incubation, the optical density was read at 595 nm, and the protein content in the sample was estimated. Finally, the dilutions of the homogenates were prepared with respect to lower protein concentrations for the enzyme inhibition studies.

#### Acetylcholinesterase assay

Twenty-five microliters of diluted mealybug homogenates (control and test) mixed with 25 µl of the reaction mixture that contains 50 µl of acetothiocholine and 50 µl of DTNB in 900 µl of assay buffer was incubated in triplicate at RT for 30 min. The AChE activity was evaluated spectrophotometrically at 410 nm in a microplate reader (SYNERGY H1 Hybrid Multi-Mode reader, Biotek) and represented as milliunits per milligram of protein (mU/mg). The AChE Kit was purchased from Abcam, UK, for AChE determination.

#### Glutathione-S-transferase assay

The total volume of 100 µl reaction mixtures was prepared by adding 10 µl of mealybug homogenates (control and test) to 75 µl of GST sample buffer, 10 µl of GST glutathione, and 5 µl of GST CDNB (initiator) in triplicate. These reaction mixtures were incubated at RT in a 96-well microplate. The enzyme kinetics were measured at the absorbance of 340 nm at 37°C for 20 min in a microplate reader with continuous mixing for 10 s after 60 s of lag time. The extinction coefficient of 0.0096 µM^−1^ for CDNB was used to calculate the glutathione S-transferase activity and represented as nanomolar per minute per milliliter of sample (nmol/min/ml). The GST Assay Kit was purchased from Cayman Chemical, USA, for GST determination.

### Scanning electron microscopy analysis of *P. lilacinus*

The treated and control samples of *P. lilacinus* after 72 and 96 h with ocimene (1,250 ppm) were fixed in 2.5% glutaraldehyde fixative solution in PB (0.1 M; pH 7.2) for 2 h, followed by washing and air drying. After that, the samples were subjected to dehydration using 30%–100% of ethanol solutions for 15–20 min each and fixed on aluminum stubs using dual-sided sticky carbon tape. Samples were then subjected to gold sputter coating (MC1000 ion sputter Hitachi, Japan) for 10 s, maintaining 10 Pa vacuum pressure. SEM images were obtained (SU 3900 Hitachi, Japan) at the appropriate resolution for surface morphological characteristics studies (Kanturski et al., 2015; Jayaram et al., 2022b).

### Statistical analysis

The mortality data of *P. lilacinus* based on the comparative toxicity of pure and combined compounds were organized. The median lethal dose (LD_50_) and other regression parameters were estimated by Probit analysis (Finney, 1971) in SPSS V.19.0. The percent mortality data for *P. lilacinus* were also determined using multivariate analysis of variance, and the mean values were determined by Tukey’s *post-hoc* test to find out the related significance between the tests. The requirements for homogeneity and normality of variance test for multiple factors and data modifications were not necessary.

## Results

### Toxicity of different compounds against *P. lilacinus*

The toxicity effect of different compounds including 1-cyclohexyl-2-pyrrolidone, camphene, cinnamaldehyde, citral, geraniol, L-carvone, L-limonene, L-menthol, β-myrcene, ocimene, α-terpinene, and β-terpinene was scrutinized, and the nymphal mortality data of second instar crawlers of *P. lilacinus* after 24, 48, 72, and 96 h of treatment are presented in **Table 1**. All the topically tested compounds exhibited toxicity effects and resulted in mortalities. Among the various compounds, L-menthol was found to be more effective (LD_50_ = 1.88 µg/insect) against *P. lilacinus* after 24 h, followed by L-limonene (LD_50_ = 2.06 µg/insect), geraniol (LD_50_ = 3.32 µg/insect), and ocimene (LD_50_ = 5.08 µg/insect) as compared to other compounds tested. Similarly, after 48 h, L-limonene showed the most toxic effect (LD_50_ = 0.92 µg/insect), preceded by L-menthol (LD_50_ = 1.16 µg/insect), geraniol (LD_50_ = 1.30 µg/insect), and cinnamaldehyde (LD_50_ = 1.78 µg/insect). Subsequently, after 72 h, L-menthol was seen to be the most effective compound (LD_50_ = 0.44 µg/insect), followed by β-myrcene (LD_50_ = 0.52 µg/insect), L-limonene (LD_50_ = 0.55 µg/insect), and geraniol (LD_50_ = 0.65 µg/insect). Finally, the compounds, such as L-limonene, β-myrcene, and ocimene, exhibited their potency (each at LD_50_ = 0.37 µg/insect) after 96 h of treatment. Also, the positive control Neem Baan (pesticide) caused the toxicity with LD_50_ values of 5.99, 3.21, 0.62, and 0.27 µg/insect for 24, 48, 72, and 96 h, respectively.

**Table 1:** Toxicity of various compounds against the second instar crawlers of *P. lilacinus*.

Compounds	Time (h)	LD_50_ (µg/insect)	CL (µg/insect)	Slope ± SE	Chi-square	*P*-value
1-Cyclohexyl-2-pyrrolidone	24	6.94	4.31–17.63	1.28 ± 0.25	0.40	0.94
	48	2.89	1.90–6.06	0.94 ± 0.20	0.07	1.00
	72	0.71	0.42–1.02	1.03 ± 0.20	0.31	0.96
	96	0.38	0.19–0.58	1.14 ± 0.22	0.68	0.89
Camphene	24	7.42	4.01–31.60	0.93 ± 0.22	0.77	0.86
	48	3.28	1.99–9.33	0.80 ± 0.20	0.11	0.99
	72	1.54	0.97–2.75	0.81 ± 0.19	0.33	0.96
	96	0.92	0.49–1.47	0.80 ± 0.19	0.28	0.96
Cinnamaldehyde	24	7.06	4.07–23.17	1.05 ± 0.23	0.58	0.90
	48	1.78	1.22–2.91	0.99 ± 0.20	1.38	0.71
	72	0.72	0.50–0.94	1.42 ± 0.22	3.47	0.33
	96	0.40	0.23–0.57	1.36 ± 0.23	2.19	0.53
Citral	24	8.37	4.60–32.47	1.05 ± 0.23	1.21	0.75
	48	4.53	2.74–12.86	0.92 ± 0.21	0.03	1.00
	72	1.77	1.23–2.86	1.01 ± 0.20	0.48	0.92
	96	0.61	0.32–0.91	0.96 ± 0.20	0.12	0.99
Geraniol	24	3.32	2.26–6.31	1.11 ± 0.21	0.34	0.95
	48	1.30	0.96–1.77	1.31 ± 0.21	1.34	0.72
	72	0.65	0.43–0.88	1.29 ± 0.21	2.06	0.56
	96	0.39	0.23–0.54	1.44 ± 0.24	2.24	0.52
L-carvone	24	5.97	3.72–14.95	1.17 ± 0.23	1.43	0.70
	48	2.74	1.94–4.61	1.18 ± 0.21	3.46	0.33
	72	1.27	0.87–1.90	1.02 ± 0.20	3.14	0.37
	96	0.68	0.36–1.03	0.92 ± 0.20	2.04	0.56
L-limonene	24	2.06	1.43–3.44	1.02 ± 0.20	1.20	0.75
	48	0.92	0.54–1.40	0.89 ± 0.20	0.31	0.96
	72	0.55	0.29–0.82	1.00 ± 0.20	0.20	0.98
	96	0.37	0.17–0.57	1.08 ± 0.21	0.23	0.97
L-menthol	24	1.88	1.35–2.88	1.14 ± 0.20	0.19	0.98
	48	1.16	0.82–1.64	1.14 ± 0.20	1.06	0.79
	72	0.44	0.21–0.67	1.04 ± 0.21	1.30	0.73
	96	0.39	0.19–0.57	1.18 ± 0.22	1.33	0.72
β-myrcene	24	8.70	4.47–44.83	0.92 ± 0.22	0.22	0.97
	48	1.98	1.32–3.55	0.92 ± 0.20	0.80	0.85
	72	0.52	0.30–0.75	1.16 ± 0.21	0.21	0.98
	96	0.37	0.22–0.51	1.54 ± 0.25	1.00	0.80
Ocimene	24	5.08	3.35–10.72	1.26 ± 0.23	1.28	0.74
	48	2.70	1.97–4.21	1.32 ± 0.21	1.69	0.64
	72	0.83	0.51–1.20	1.00 ± 0.20	2.27	0.52
	96	0.37	0.16–0.57	1.03 ± 0.21	0.45	0.93
α-Terpinene	24	8.30	4.26–44.14	0.89 ± 0.22	0.02	1.00
	48	2.17	1.47–3.83	0.97 ± 0.20	0.74	0.87
	72	0.97	0.67–1.35	1.15 ± 0.20	0.57	0.90
	96	0.53	0.31–0.75	1.19 ± 0.21	1.01	0.80
β-Terpinene	24	9.93	5.01–53.63	0.96 ± 0.23	0.29	0.96
	48	2.87	1.56–14.07	0.61 ± 0.19	0.02	1.00
	72	1.41	0.67–3.54	0.58 ± 0.19	0.13	0.99
	96	0.80	0.32–1.35	0.69 ± 0.19	0.13	0.99
Neem Baan (Azadirachtin 1,500 ppm)	24	5.99	3.26–25.93	0.49 ± 0.21	0.49	0.92
	48	3.21	1.80–13.23	0.67 ± 0.19	0.43	0.94
	72	0.62	0.07–1.27	0.51 ± 0.19	0.01	1.00
	96	0.27	0.02–0.57	0.63 ± 0.01	0.77	0.86

### Combination of different compounds against *P. lilacinus*

The combination of different compounds, viz., ocimene + β-myrcene, ocimene + L-limonene, ocimene + geraniol, ocimene + L-menthol, β-myrcene + L-limonene, β-myrcene + geraniol, β-myrcene + L-menthol, L-limonene + geraniol, and geraniol + L-menthol, at a 1:1 ratio of their 96 h LD_50_ concentration was tested for the toxicity and synergistic activity against the second instar crawlers of *P. lilacinus* between 24- and 96-h time intervals, and the results are shown in **Table 2**. All the tested combinations against *P. lilacinus* revealed toxicity and synergistic effects. Among the multiple combinations, geraniol + L-menthol showed a significant effect (LD_50_ = 0.29 µg/insect), followed by L-limonene + geraniol (LD_50_ = 0.35 µg/insect), ocimene + geraniol (LD_50_ = 0.87 µg/insect), and β-myrcene + L-limonene (LD_50_ = 0.97 µg/insect) after 24 h. Subsequently, after 48 h, geraniol + L-menthol was found to be a more effective combination (LD_50_ = 0.10 µg/insect), preceded by L-limonene + geraniol (LD_50_ = 0.13 µg/insect), β-myrcene + geraniol (LD_50_ = 0.33 µg/insect), and ocimene + geraniol (LD_50_ = 0.42 µg/insect). Furthermore, 72-h treatment showed that geraniol + L-menthol had a significant level of toxicity (LD_50_ = 0.05 µg/insect), followed by L-limonene + geraniol (LD_50_ = 0.08 µg/insect), β-myrcene + geraniol (LD_50_ = 0.18 µg/insect), and ocimene + L-limonene (LD_50_ = 0.24 µg/insect). Eventually, the combinations such as geraniol + L-menthol and L-limonene + geraniol at 96 h showed remarkable toxicity efficiency (each at LD_50_ = 0.03 µg/insect).

**Table 2:** Toxicity of various combined compounds against the second instar crawlers of *P. lilacinus*.

Compounds	Time (h)	LD_50_ (µg/insect)	CL (µg/insect)	Slope ± SE	Chi-square	*p*-value	Cotoxicity coefficient	Interaction type
Ocimene+ β-myrcene	24	1.63	0.60–41.55	0.89 ± 0.25	0.28	0.97	311.66	Synergistic
	48	0.56	0.24–14.49	0.60 ± 0.20	0.01	1.00	482.14	Synergistic
	72	0.26	0.14–1.56	0.61 ± 0.19	0.02	1.00	319.23	Synergistic
	96	0.10	0.03–0.50	0.45 ± 0.19	0.03	1.00	370.00	Synergistic
Ocimene+ L-limonene	24	1.02	0.50–6.10	1.16 ± 0.27	0.49	0.92	498.04	Synergistic
	48	0.50	0.27–2.00	0.91 ± 0.21	0.36	0.95	540.00	Synergistic
	72	0.24	0.15–0.70	0.79 ± 0.20	0.29	0.96	345.83	Synergistic
	96	0.13	0.07–0.26	0.72 ± 0.19	0.25	0.97	284.62	Synergistic
Ocimene+ Geraniol	24	0.87	0.45–4.14	1.13 ± 0.25	0.25	0.97	583.91	Synergistic
	48	0.42	0.25–1.26	0.98 ± 0.21	0.76	0.86	642.86	Synergistic
	72	0.31	0.17–1.31	0.73 ± 0.20	0.60	0.90	267.74	Synergistic
	96	0.14	0.08–0.31	0.70 ± 0.19	1.80	0.62	264.29	Synergistic
Ocimene+ L-menthol	24	1.51	0.59–25.76	0.93 ± 0.25	0.08	0.99	336.42	Synergistic
	48	0.75	0.32–12.10	0.70 ± 0.21	0.12	0.99	360.00	Synergistic
	72	0.55	0.23–31.10	0.55 ± 0.20	0.01	1.00	150.91	Synergistic
	96	0.15	0.06–3.71	0.44 ± 0.19	0.10	0.99	246.67	Synergistic
β-myrcene+ L-limonene	24	0.97	0.49–5.05	1.20 ± 0.27	0.15	0.99	896.91	Synergistic
	48	0.66	0.29–10.63	0.68 ± 0.20	0.06	1.00	300.00	Synergistic
	72	0.38	0.16–91.80	0.47 ± 0.19	0.02	1.00	136.84	Synergistic
	96	0.11	0.06–0.22	0.66 ± 0.19	0.19	0.98	336.36	Synergistic
β-myrcene+ Geraniol	24	1.47	0.52–60.41	0.74 ± 0.22	0.16	0.98	591.84	Synergistic
	48	0.33	0.19–1.24	0.78 ± 0.20	0.28	0.96	600.00	Synergistic
	72	0.18	0.11–0.40	0.80 ± 0.19	0.43	0.94	288.89	Synergistic
	96	0.10	0.06–0.16	0.91 ± 0.19	0.31	0.96	370.00	Synergistic
β-myrcene+ L-menthol	24	1.70	0.58–72.25	0.78 ± 0.23	0.27	0.97	511.76	Synergistic
	48	0.96	0.37–27.63	0.68 ± 0.21	0.24	0.97	206.25	Synergistic
	72	0.45	0.22–5.70	0.63 ± 0.20	0.06	1.00	115.56	Synergistic
	96	0.24	0.12–3.47	0.51 ± 0.19	0.01	1.00	154.17	Synergistic
L-limonene+ Geraniol	24	0.35	0.20–1.38	0.78 ± 0.20	0.52	0.91	588.57	Synergistic
	48	0.13	0.08–0.22	0.88 ± 0.19	1.19	0.76	707.69	Synergistic
	72	0.08	0.05–0.11	0.95 ± 0.20	1.15	0.77	687.50	Synergistic
	96	0.03	0.01–0.05	0.86 ± 0.20	0.61	0.90	1233.33	Synergistic
Geraniol+L-menthol	24	0.29	0.17–0.85	0.83 ± 0.20	0.28	0.96	1144.83	Synergistic
	48	0.10	0.06–0.18	0.77 ± 0.19	0.21	0.98	1300.00	Synergistic
	72	0.05	0.02–0.08	0.73 ± 0.19	0.10	0.99	1300.00	Synergistic
	96	0.03	0.01–0.05	0.71 ± 0.20	0.05	1.00	1300.00	Synergistic

### Percent mortality of different compounds against *P. lilacinus*

The toxicity of different compounds and concentrations against *P. lilacinus* reflected in the percent mortality after 24, 48, 72, and 96 h is summarized in **Tables 3**–**6**. The pooled mean (± SE) percent mortality was found to be significantly different across the compounds (*F*_11, 299_ = 18.44, 12.45, 11.58, and 9.92) and concentrations (*F*_4, 299_ = 170.29, 189.09, 159.28, and 138.44) with *p* < 0.0001 for 24, 48, 72, and 96 h, respectively. However, the mean percent mortality was not significantly different (*p* > 0.05) between the interaction of compounds and concentrations. Results showed that, among different compounds, the pooled mean mortality was significantly higher in L-menthol (42.80 ± 19.48), L-limonene (54.40 ± 16.60), L-menthol (66.40 ± 17.05), and β-myrcene (74.80 ± 19.82) after 24, 48, 72, and 96 h, respectively, in comparison to other tested compounds. The pooled mean mortality was higher in the highest concentration at 5,000 ppm (48.33 ± 1.17, 66.67 ± 1.27, 81.17 ± 1.43, and 88.33 ± 1.46 for 24, 48, 72, and 96 h, respectively) as compared to other concentrations. The overall percent mortality was superior in L-menthol (70 ± 7.07), geraniol (82 ± 8.37), cinnamaldehyde (94 ± 5.48), and geraniol and β-myrcene (each at 98 ± 4.47) against *P. lilacinus* after 24, 48, 72, and 96 h, respectively, in contrast to the other remaining compounds.

**Table 3:** Toxicity effect of various compounds against the second instar crawlers of *P. lilacinus*.

	Percent mortality (mean ± SE) at different concentrations after 24 h					
Compounds	5,000 ppm	2,500 ppm	1,250 ppm	625 ppm	312.5 ppm	Pooled mean
1-Cyclohexyl-2-pyrrolidone	42 ± 8.37	28 ± 8.37	20 ± 12.25	8 ± 8.37	4 ± 5.48	20.40 ± 16.20 d
Camphene	44 ± 8.94	30 ± 15.81	26 ± 8.94	18 ± 8.37	8 ± 8.37	25.20 ± 15.58 cd
Cinnamaldehyde	44 ± 8.94	30 ± 7.07	22 ± 13.04	16 ± 5.48	6 ± 5.48	23.60 ± 15.24 d
Citral	38 ± 8.37	30 ± 15.81	22 ± 8.37	14 ± 5.48	4 ± 5.48	21.60 ± 14.91 d
Geraniol	60 ± 12.25	42 ± 17.89	32 ± 8.37	20 ± 10.00	14 ± 5.48	33.60 ± 19.76 bc
L-carvone	50 ± 10.00	28 ± 8.37	20 ± 7.07	16 ± 8.94	6 ± 5.48	24.00 ± 16.83 d
L-Limonene	62 ± 8.37	56 ± 5.48	46 ± 5.48	26 ± 5.48	20 ± 7.07	42.00 ± 17.79 ab
L-menthol	70 ± 7.07	54 ± 8.94	42 ± 8.37	28 ± 4.47	20 ± 7.07	42.80 ± 19.48 a
β-myrcene	40 ± 12.25	32 ± 8.37	22 ± 4.47	16 ± 5.48	8 ± 8.37	23.60 ± 13.81 d
Ocimene	50 ± 15.81	32 ± 10.95	24 ± 13.42	16 ± 5.48	4 ± 5.48	25.20 ± 18.73 cd
α-Terpinene	42 ± 4.47	32 ± 8.37	24 ± 8.94	16 ± 8.94	10 ± 10.00	24.80 ± 13.88 d
β-Terpinene	38 ± 4.47	28 ± 13.04	20 ± 10.00	14 ± 5.48	6 ± 8.94	21.20 ± 13.94 d
Pooled mean	48.33 ± 1.17 a	35.17 ± 1.17 b	26.67 ± 1.17 c	17.33 ± 1.17 d	9.17 ± 1.17 e	
Compounds	*F*_11, 299_ = 18.44; *p* < 0.0001					
Concentrations	*F*_4, 299_ = 170.29; *p* < 0.0001					
Compound × Concentrations	*F*_44, 299_ = 0.69; *p* > 0.05					

Mean of five replications: Means followed by the same letters within a column are not statistically significant (p > 0.05) by Tukey’s HSD.

**Table 4:** Toxicity effect of various compounds against the second instar crawlers of *P. lilacinus*.

	Percent mortality (mean ± SE) at different concentrations after 48 h					
Compounds	5,000 ppm	2,500 ppm	1,250 ppm	625 ppm	312.5 ppm	Pooled mean
1-Cyclohexyl-2-pyrrolidone	58 ± 8.37	48 ± 4.47	38 ± 10.95	26 ± 5.48	18 ± 4.47	37.60 ± 16.15 def
Camphene	56 ± 8.94	46 ± 11.40	36 ± 11.40	30 ± 10.00	20 ± 7.07	37.60 ± 15.62 def
Cinnamaldehyde	72 ± 8.37	50 ± 7.07	42 ± 8.37	34 ± 5.48	24 ± 8.94	44.40 ± 18.05 bcd
Citral	52 ± 8.37	40 ± 14.14	30 ± 12.25	22 ± 13.04	14 ± 5.48	31.60 ± 17.00 f
Geraniol	82 ± 8.37	60 ± 7.07	48 ± 13.04	32 ± 4.47	24 ± 5.48	49.20 ± 22.35 abc
L-carvone	70 ± 14.14	40 ± 7.07	30 ± 7.07	24 ± 5.48	16 ± 5.48	36.00 ± 20.62 def
L-limonene	76 ± 11.40	62 ± 4.47	56 ± 8.94	44 ± 5.48	34 ± 8.94	54.40 ± 16.60 a
L-menthol	80 ± 7.07	60 ± 10.00	52 ± 4.47	36 ± 5.48	28 ± 13.04	51.20 ± 20.27 ab
β-myrcene	68 ± 8.37	50 ± 10.00	40 ± 12.25	34 ± 5.48	24 ± 11.40	43.20 ± 17.73 bcde
Ocimene	68 ± 16.43	42 ± 8.37	32 ± 13.04	24 ± 5.48	10 ± 10.00	35.20 ± 22.38 ef
α-terpinene	62 ± 10.95	54 ± 16.73	40 ± 10.00	34 ± 11.40	18 ± 8.37	41.60 ± 19.08 cde
β-terpinene	56 ± 13.42	48 ± 8.37	42 ± 16.43	34 ± 15.17	28 ± 10.95	41.60 ± 15.73 cde
Pooled mean	66.67 ± 1.27 a	50 ± 1.27 b	40.50 ± 1.27 c	31.17 ± 1.27 d	21.50 ± 1.27 e	
Compounds	*F*_11, 299_ = 12.45; *p* < 0.0001					
Concentrations	*F*_4, 299_ = 189.09; *p* < 0.0001					
Compound × Concentrations	*F*_44, 299_ = 0.93; *p* > 0.05					

Mean of five replications: Means followed by the same letters within a column are not statistically significant (p > 0.05) by Tukey’s HSD.

**Table 5:** Toxicity effect of various compounds against the second instar crawlers of *P. lilacinus*.

	Percent mortality (mean ± SE) at different concentrations after 72 h					
Compounds	5,000 ppm	2,500 ppm	1,250 ppm	625 ppm	312.5 ppm	Pooled mean
1-Cyclohexyl-2-pyrrolidone	82 ± 13.04	72 ± 4.47	58 ± 4.47	46 ± 13.42	38 ± 4.47	59.20 ± 18.47 abc
Camphene	68 ± 17.89	56 ± 15.17	44 ± 13.42	38 ±18.37	30 ± 7.07	47.20 ± 18.15 de
Cinnamaldehyde	94 ± 5.48	74 ± 13.42	58 ± 10.95	44 ± 13.42	36 ± 8.94	61.20 ± 23.50 ab
Citral	68 ± 14.83	58 ± 14.83	40 ± 7.07	32 ± 13.04	24 ± 5.48	44.40 ± 19.81 e
Geraniol	92 ± 8.37	72 ± 8.37	62 ± 8.37	50 ± 7.07	36 ± 5.48	62.40 ± 20.67 ab
L-carvone	80 ± 14.14	54 ± 8.94	46 ± 5.48	38 ± 8.37	30 ± 12.25	49.60 ± 19.89 cde
L-limonene	84 ± 13.42	74 ± 5.48	64 ± 8.94	50 ± 10.00	42 ± 8.37	62.80 ± 17.92 ab
L-menthol	90 ± 7.07	74 ± 8.94	66 ± 5.48	56 ± 5.48	46 ± 11.40	66.40 ± 17.05 a
β-myrcene	86 ± 11.40	80 ± 7.07	68 ± 8.37	52 ± 8.37	40 ± 10.00	65.20 ± 19.39 a
Ocimene	84 ± 8.94	62 ± 13.04	54 ± 11.40	46 ± 5.48	36 ± 13.42	56.40 ± 19.34 abcd
α-terpinene	82 ± 10.95	66 ± 16.73	52 ± 10.95	42 ± 17.89	30 ± 10.00	54.40 ± 22.38 bcde
β-terpinene	64 ± 19.49	54 ± 11.40	48 ± 13.04	42 ± 14.83	36 ± 18.17	48.80 ± 17.40 de
Pooled mean	81.17 ± 1.43 a	66.33 ± 1.43 b	55.00 ± 1.43 c	44.67 ± 1.43 d	35.33 ± 1.43 e	
Compounds	*F*_11, 299_ = 11.58; *p* < 0.0001					
Concentrations	*F*_4, 299_ = 159.28; *p* < 0.0001					
Compound × Concentrations	*F*_44, 299_ = 0.65; *p* > 0.05					

Mean of five replications: Means followed by the same letters within a column are not statistically significant (p > 0.05) by Tukey’s HSD.

**Table 6:** Toxicity effect of various compounds against the second instar crawlers of *P. lilacinus*.

	Percent mortality (mean ± SE) at different concentrations after 96 h					
Compounds	5,000 ppm	2,500 ppm	1,250 ppm	625 ppm	312.5 ppm	Pooled mean
1-Cyclohexyl-2-pyrrolidone	92 ± 8.37	80 ± 7.07	70 ± 7.07	62 ± 13.04	46 ± 8.94	70.00 ± 18.03 ab
Camphene	74 ± 19.49	62 ± 21.68	52 ± 8.37	46 ± 11.40	36 ± 13.42	54.00 ± 19.58 e
Cinnamaldehyde	96 ± 5.48	86 ± 8.94	68 ± 13.04	60 ± 10.00	48 ± 4.47	71.60 ± 19.51 a
Citral	82 ± 20.49	72 ± 13.04	60 ± 12.25	50 ± 12.25	40 ± 12.25	60.80 ± 20.19 bcde
Geraniol	98 ± 4.47	84 ± 8.94	74 ± 8.94	64 ± 11.40	46 ± 8.94	73.20 ± 19.73 a
L-carvone	84 ± 8.94	64 ± 16.73	56 ± 5.48	50 ± 7.07	40 ± 14.14	58.80 ± 18.33 cde
L-limonene	90 ± 10.00	80 ± 10.00	72 ± 4.47	58 ± 8.37	48 ± 10.95	69.60 ± 17.44 ab
L-menthol	94 ± 5.48	80 ± 7.07	70 ± 7.07	60 ± 7.07	48 ± 10.95	70.40 ± 17.67 ab
β-myrcene	98 ± 4.47	88 ± 4.47	78 ± 8.37	62 ± 13.04	48 ± 8.37	74.80 ± 19.82 a
Ocimene	90 ± 10.00	78 ± 10.95	70 ± 7.07	60 ± 12.25	48 ± 8.37	69.20 ± 17.30 abc
α-terpinene	90 ± 12.25	78 ± 21.68	62 ± 4.47	56 ± 11.40	40 ± 12.25	65.20 ± 21.63 abcd
β-terpinene	72 ± 16.43	62 ± 10.95	56 ± 8.94	46 ± 19.49	40 ± 15.81	55.20 ± 17.82 de
Pooled mean	88.33 ± 1.46 a	76.17 ± 1.46 b	65.67 ± 1.46 c	56.17 ± 1.46 d	44.00 ± 1.46 e	
Compounds	*F*_11, 299_ = 9.92; *p* < 0.0001					
Concentrations	*F*_4, 299_ = 138.44; *p* < 0.0001					
Compound × Concentrations	*F*_44, 299_ = 0.33; *p* > 0.05					

Mean of five replications: Means followed by the same letters within a column are not statistically significant (p > 0.05) by Tukey’s HSD.

### Detoxification enzyme activities of ocimene and myrcene against *P. lilacinus*

**Figure 2:**
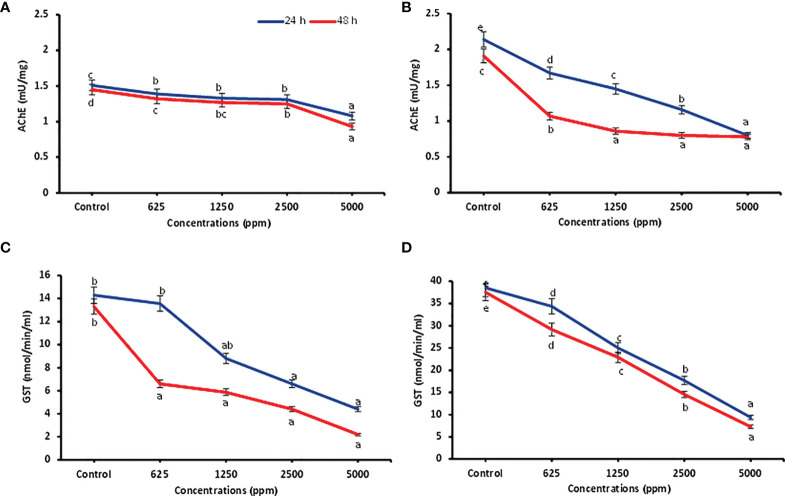
Detoxification enzyme inhibition activities. AChE inhibition in *P. lilacinus* treated with ocimene **(A)** and β-myrcene **(B)**. GST inhibition in *P. lilacinus* treated with ocimene **(C)** and β-myrcene **(D)**. Bars represent the standard error ( ± SE) of three replications. Means followed by the same letters within a column do not differ significantly by Tukey’s HSD test (p > 0.05).

Detoxifying enzyme inhibition activities of ocimene and β-myrcene (based on activity and feasibility) were carried out against *P. lilacinus* after 24 and 48 h of treatment and are presented in **Figure 2**. Data showed that all the concentrations of ocimene significantly inhibited the AChE enzyme after 24 and 48 h (*F*_4,14_ = 69.39; *p* < 0.0001 and *F*_4,14_ = 221.70; *p* < 0.0001) in *P. lilacinus*, as compared to the control. Similarly, all the concentrations of β-myrcene highly inhibited the AChE activity (*F*_4,14_ = 941.73; *p* < 0.0001 and *F*_4,14_ = 443.20; *p* < 0.0001), when compared to the control after 24 and 48 h. Among different concentrations, ocimene at 5,000 ppm reported significantly higher inhibition of AChE after 24 and 48 h of treatment (1.08 ± 0.01 and 0.93 ± 0.01 mU/mg, respectively), followed by 2,500 ppm (1.31 ± 0.03 and 1.25 ± 0.01 mU/mg) as compared to other lower concentrations (625–1,250 ppm) (**Figure 2A**). Similarly, β-myrcene at 5,000 ppm revealed higher inhibition of AChE activity after 24 and 48 h (0.80 ± 0.01 and 0.78 ± 0.02 mU/mg, respectively), followed by 2,500 ppm (1.16 ± 0.02 and 0.80 ± 0.02 mU/mg), as compared to other lower concentrations (**Figure 2B**). Correspondingly, for the GST assay, all the concentrations of ocimene after 24 and 48 h also substantially inhibited the GST activity (*F*_4,14_ = 13.32; *p* < 0.003 and *F*_4,14_ = 14.89; *p* < 0.0001) as compared to the control. Similarly, all the concentrations of β-myrcene significantly inhibited the GST activity (*F*_4,14_ = 195.04; *p* < 0.0001 and *F*_4,14_ = 141.74; *p* < 0.0001) in contrast to the control. Among concentrations, ocimene at 5,000 ppm exhibited higher inhibition of GST after 24 and 48 h (4.40 ± 1.68 and 2.19 ± 0.64 mU/mg, respectively) and was preceded by 2,500 ppm (6.60 ± 0.64 and 4.40 ± 0.64 mU/mg) than at lower concentrations (625–1,250 ppm) (**Figure 2C**). Likewise, β-myrcene at 5,000 ppm showed higher inhibition of GST after 24 and 48 h (9.37 ± 0.92 and 7.29 ± 1.20 mU/mg, respectively), followed by 2,500 ppm (17.70 ± 0.92 and 14.58 ± 0.92 mU/mg), when compared to lower concentrations (**Figure 2D**).

### Scanning electron microscopy analysis of *P. lilacinus*

**Figure 3:**
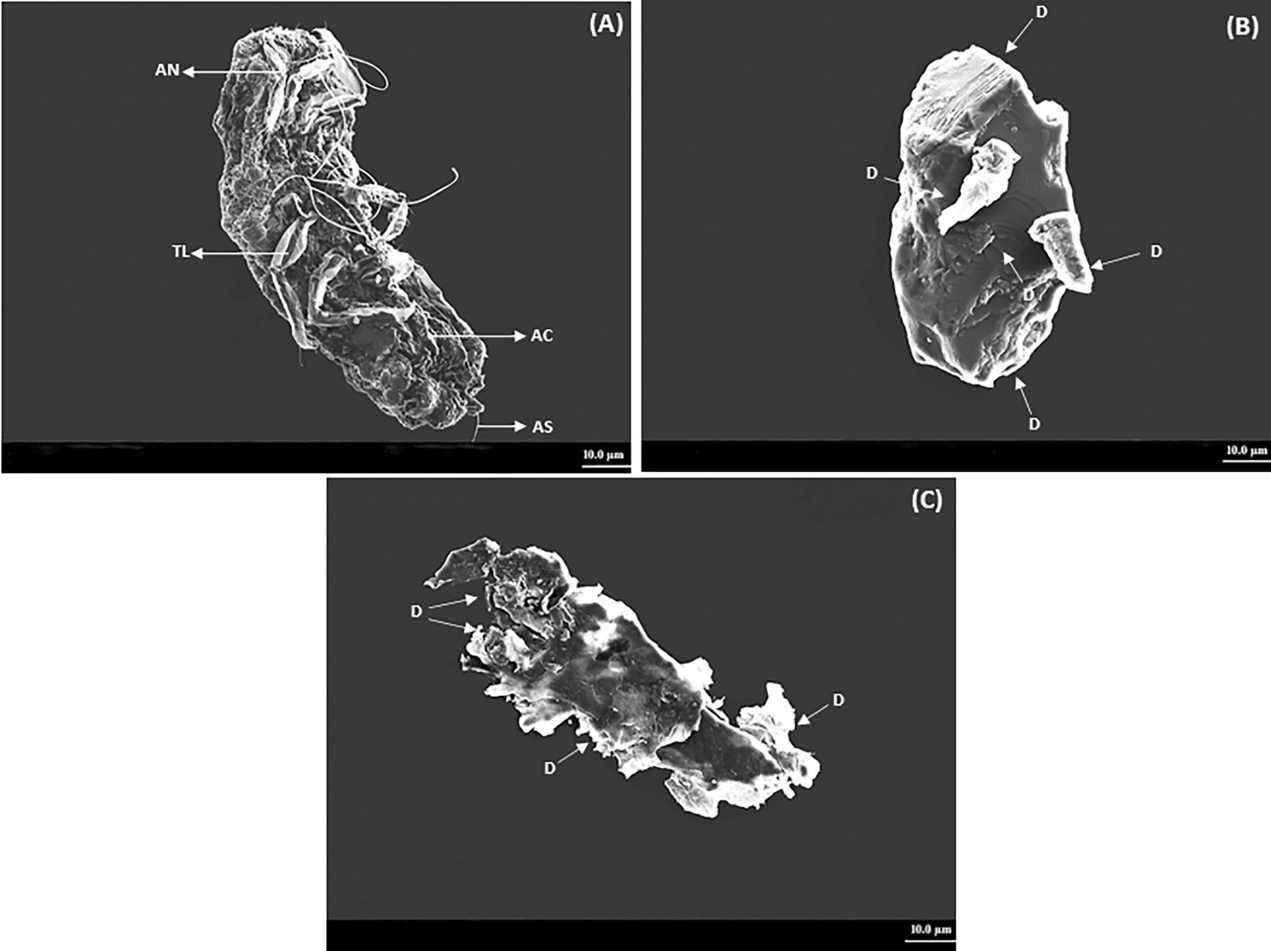
SEM analysis on deformities observed in *P. lilacinus* treated with ocimene. **(A)** Control, **(B)** 72 h after treatment, and **(C)** 96 h after treatment. AN, antenna; TL,thoracic leg; AC,abdominal cuticle; AS, abdominal setae; D, deformity or disintegration.

The crawlers of *P. lilacinus* topically treated with the ocimene altered the motility and eventually caused mortality. The treated *P. lilacinus* exhibited several deformities on the abdominal cuticle, setae, and thoracic leg after 72 and 96 h of treatment. The *P. lilacinus* treated with ocimene after 72 and 96 h showed a color change from pale yellow to dark brown with the accumulation of body fluids and thick encrustations on the external surface. SEM experiments showed that the crawlers of *P. lilacinus* with ocimene resulted in multiple habitus symptoms based on the time interval given in **Figures 3A–C**. **Figure 3A** represents the untreated *P. lilacinus* (control) with the distinct structure of cuticle, legs, and setae without any deformities after 96 h of treatment, whereas the severe leg deformities, lack of setae, and the abdominal cuticular damages were evidenced after 72 h (**Figure 3B**). *P. lilacinus* showed complete disintegration of external habitus after 96 h (**Figure 3C**) as compared to the control.

## Discussion

The present study aims to apply phytochemicals to control the mealybugs that are safer, more eco-friendly, and more effective than chemical pesticides (Regnault-Roger et al., 2012; Isman, 2016). In this study, toxicity and synergistic and detoxification enzyme inhibition activities of some plant-based active compounds and their combinations against the crawlers of *P. lilacinus* were discussed. Many of these phytochemicals are derived from the essential oils that exhibited the insecticidal activities reported earlier (Bakkali et al., 2008). In our study, phytochemicals such as 1-cyclohexyl-2-pyrrolidone, camphene, cinnamaldehyde, citral, geraniol, L-carvone, L-limonene, L-menthol, β-myrcene, ocimene, α-terpinene, and β-terpinene have been tested to belong to the chemical class of monoterpenoids, terpenoids, and phenylpropanoids, among others. Essential oils are constituted by a mixture of compounds predominantly by monoterpenes with promising insecticidal activities (Park et al., 2017). For example, the monoterpenoid pure compounds, such as thymol, carveol, and cymene, and other compounds from the plant essential oils exhibited effective contact toxicity against *Blattella germanica* (Yeom et al., 2012). Likewise, carvone exhibited the highest contact toxicity to *Tribolium castaneum* and *Sitophilus oryzae* (Abdelgaleil et al., 2009). Almost all the tested compounds in the present study showed insecticidal activity against *P. lilacinus*. Among the various compounds tested, the high insecticidal efficiency was observed in the monoterpenes of ocimene and β-myrcene (each at LD_50_ = 0.37 µg/insect). A previous study also reported that such pure compounds from the essential oil of *Tagetes minuta* showed residual toxicity, repellent, feeding deterrent, growth (feeding) inhibition, and weight reduction activities against the diamondback moth, *Plutella xylostella* (Reddy et al., 2016).

Many essential oil pure compounds of monoterpenes (carvacrol) and sesquiterpenes (α-bisabolol and chamazulene) from various plant sources have been reported for their insecticidal activities against various pest insects (Rodríguez et al., 2005; Tang et al., 2011; Nenaah, 2014). The contact toxicity experiments on *P. lilacinus* revealed that monoterpenes of ocimene and β-myrcene were the active pure compounds that cause insecticidal activity compared to other compounds in the present study. Ocimene is found in several plant species, such as *Ocimum gratissimum*, *Evodia lenticellata*, *Acorus calamus*, and *Aegle marmelos*, and has been reported to have contact toxicity against *Callosobruchus chinensis*, *Lasioderma serricorne*, *Liposcelis bostrychophila, Oryzaephilus surinamensis*, *Rhyzopertha dominica*, *S. oryzae*, and *T. castaneum* (Ogendo et al., 2008; Cao et al., 2018). β-myrcene has been found in many plants, namely, *Aegle marmelos and Peucedanum terebinthinaceum*, and has been stated to have contact toxicity against *L. serricorne*, *L. bostrychophila, S. oryzae*, and *T. castaneum* (Abdelgaleil et al., 2009; Sun et al., 2020). These findings strongly suggested the occurrence of contact toxicity property of pure compounds from the essential oils of several plants against various insect pests.

Insecticidal activities of pure compounds from plant essential oils depend on the nature of the compound, tested concentration, selected stage, and type of insect (Mathela et al., 1994; Lee et al., 2001; Tripathi et al., 2001). Generally, the biological activities of plant essential oils often result from the synergistic nature of their varied active components that can be used individually or as a mixture for pest control (Anaya-Eugenio et al., 2016; Afshar et al., 2017). In our study, the comparative toxicity assay using different compounds by the topical application method against the second instar crawlers of *P. lilacinus* revealed that the compounds, namely, L-limonene, β-myrcene, and ocimene, were found to be most effective against *P. lilacinus* (each at LD_50_ = 0.37 µg/insect) after 96 h of treatment. Similar findings were reported earlier with the pure compounds of pulegone and menthofuran from *Minthostachys verticillata* against *P. ficus* (Peschiutta et al., 2017). Eventually, the different combinations of pure compounds screened against *P. lilacinus* revealed that the combinations, namely, geraniol + L-menthol and L-limonene + geraniol, were found to be most effective against *P. lilacinus* (each at LD_50_ = 0.03 µg/insect) after 96 h of treatment. Our study indicated that all the tested combinations of the pure compounds resulted in the synergistic activity for topical toxicity against *P. lilacinus*. However, the present study attempted for the first time to study the combined actions of pure compounds of essential oils against *P. lilacinus*. The primary components of plant essential oils are blended to form a new formulation (binary mixtures) possessing additive or synergistic properties upon toxicity (Feitosa-Alcantara et al., 2017). The current findings of our study coincided with the synergistic activity of the major compounds of the essential oil from *Artemisia absinthium*, namely, α-bisabolol, carvacrol, and chamazulene, at a 1:1:1 ratio against *Diaphorina citri* (Rizvi et al., 2018). It is comparatively suggested that the binary mixtures of pure compounds rather than individual compounds can be helpful since they possess numerous mechanisms of action and may stall the emergence of resistance in pests.

Generally, the botanicals exhibit potent insecticidal activities by directing the neuro-endocrine system and metabolism of the target insects reported earlier (Parthiban et al., 2020; Ramachandran et al., 2022). Therefore, before exploratory findings, several studies have consequently documented the suggested alterations in various biochemical components of target insects exposed to various botanicals (Shekari et al., 2008). The prominent detoxification enzymes such as AChE, carboxyl esterase, cytochrome P450 monooxygenases, and GST enable the surface-feeding insects to retain their biological roles by detoxifying the toxic chemicals (xenobiotics), including insecticides and harmful secondary metabolites that originated from the host plants (Oakeshott et al., 2005; Vashist and Ahmad, 2011; Feyereisen, 2012). The biochemical changes may be linked to the lethal effect of applied botanicals, resulting in insect physicochemical process interruption (Parthiban et al., 2020). The pure compounds of essential oils follow a number of mechanisms against the insect pests, including the inhibition of GST and cytochrome P450 monooxygenase, digestive enzymes, growth, and neurotoxicity (Park and Tak, 2016). Most of the essential oils are constituted by monoterpenes that follow neurotoxicity as the primary mode of action observed by the symptoms against the biological control of various insects reported earlier (Kostyukovsky et al., 2002; Priestley et al., 2003; Bullangpoti et al., 2012; Yeom et al., 2012).

Among the various detoxifying enzymes, AChE and GST are the primary target enzymes of various researchers to study the biochemical changes in insects (Koodalingam et al., 2011; Shojaei et al., 2017). AChE and GST are familiar because of their crucial role in metabolism, physiological activities, and detoxification processes (Parthiban et al., 2020). Hence, in the present study, AChE and GST were chosen for their actions in *P. lilacinus* fed with tea leaves and topically treated at different concentrations of pure compounds, namely, ocimene and β-myrcene (monoterpenes), which significantly inhibited the enzyme activity for 24 and 48 h after treatment compared to control mealybugs. AChE inhibition is generally involved in the accretion of acetylcholine (ACh) in cholinergic synapses that result in the higher modulation of the cholinergic system (Boyer et al., 2012). Subsequently, GST catalyzes the synthetic pesticides usually later in the phase I metabolic process (Kranthi, 2005). The current findings were supported by the results of Dolma et al. (2021), stating that *T. minuta* oil inhibited the AChE and GST activity against *P. xylostella*. Similarly, the essential oil from *Artemisia maritima* inhibited the GST activity against *C. chinensis* and *C. maculatus*, as reported previously (Chauhan et al., 2022). However, pure compounds of monoterpenes such as α-bisabolol, 1-8-cineole, carvone, carvacrol, chamazulene, and limonene decreased the AChE and GST activities in insects (Abdelgaleil et al., 2009; Rizvi et al., 2018).

The integument of insects is broadly classified into a hydrophilic layer (endocuticle) composed of proteins and lipids and a lipophilic layer (epi/endocuticle) constituted by chitin (Yu, 2008). It is well-known that the polar terpenoid compounds improve the entry of hydrophilic drugs, while non-polar terpenoid compounds facilitate the uptake of lipophilic medicines (Tak and Isman, 2017). Similar to this, the monoterpenoid compounds of essential oils frequently exhibit a variety of hydrophilic–hydrophobic characteristics that allow them to readily pass insect cuticles and affect their physiological processes (López and Pascual-Villalobos, 2010; Tak et al., 2016). These findings also suggested that monoterpenes ocimene and β-myrcene followed the same mode of action to arrest the physiological processes in *P. lilacinus*.

SEM studies revealed that the ocimene caused significant structural changes in the abdominal cuticle, setae, and thoracic leg of *P. lilacinus*, also discussed here. Due to the lack of literature about the effect of pure compounds of essential oils against *P. lilacinus*, the SEM findings were not compared to the earlier studies. However, few studies on the structural deformities caused by *T. minuta* oil against *A. craccivora* (Jayaram et al., 2022b) and *P. xylostella* (Dolma et al., 2021) were reported earlier, which strongly supported the current findings of the present study. Sahu et al. (2021) reported that some pure compounds, namely, diallyl disulfide and eucalyptol, resulted in severe damage to the elytra against *S. oryzae*. It is suggested that the pure phytochemical compounds lead to scales injury, impairment of setae, and disintegration of the epicuticular layer that results in cuticle abrasion and desiccation (Sahu et al., 2021). The penetration of the eucalyptol from rosemary essential oil topically applied against *Trichoplusia ni* was also evidenced earlier (Tak and Isman, 2017).

The present investigation concluded that *P. lilacinus* can be controlled using the pure compounds from essential oils of various plant sources. The toxicity of subjected individual pure compounds or their combinations may be the cause of insecticidal activity. The effectiveness of pure compounds against *P. lilacinus* has not been previously described, according to the literature review. As a result, this investigation is a novel approach and has never been done before to examine the toxicity of pure compounds from the essential oils against *P. lilacinus*.

## Data availability statement

The original contributions presented in the study are included in the article/supplementary material. Further inquiries can be directed to the corresponding author.

## Author contributions

CA performed the experiments, data curation, formal analysis, validation, and writing the original draft. KB assisted with the experiments. SR contributed to conceptualization, supervision, funding acquisition, formal analysis, and writing—review and editing. All authors contributed to the article and approved the submitted version.

## Funding

This research was supported and funded by the Council of Scientific and Industrial Research (CSIR), New Delhi, granting the proposal “CSIR Aroma Mission” HCP-0007 (Grant No. 33/Mission/Aroma/2016-MD). IHBT communication number for this article is 5130.

## Conflict of interest

The authors declare that the research was conducted in the absence of any commercial or financial relationships that could be construed as a potential conflict of interest.

## Publisher’s note

All claims expressed in this article are solely those of the authors and do not necessarily represent those of their affiliated organizations, or those of the publisher, the editors and the reviewers. Any product that may be evaluated in this article, or claim that may be made by its manufacturer, is not guaranteed or endorsed by the publisher.
